# Herpesvirus Diversity in Atlantic Procellariiformes

**DOI:** 10.3390/vetsci12121143

**Published:** 2025-11-29

**Authors:** Laura Baes, Carolina Reigada, Aricia Duarte-Benvenuto, Roberta Zamana Ramblas, Carlos Sacristán, Juliana Mariotti Guerra, Thais Marcondes Schreiner, Rafael Sardinha Murro, José Luiz Catão-Dias, Ana Carolina Ewbank

**Affiliations:** 1 Programa de Pós-graduação em Ecologia e Recursos Naturais, Universidade Federal de São Carlos, São Carlos 139565-905, Brazil; 2 Laboratório de Ecologia de Interações, Departamento de Ecologia e Biologia Evolutiva, Universidade Federal de São Carlos, São Carlos 139565-905, Brazil; 3 Laboratório de Patologia Comparada de Animais Selvagens (LAPCOM), Faculdade de Medicina Veterinária e Zootecnia, Universidade de São Paulo, São Paulo 05508-900, Brazil; 4 Centro de Investigación en Sanidad Animal (CISA-INIA), CSIC, 28130 Valdeolmos, Madrid, Spain; 5 Instituto de Pesquisas Cananéia (IPeC), Cananéia 11990-000, Brazil

**Keywords:** alphaherpesvirus, *Mardivirus*, Diomedeidae, Procelariidae, seabirds, virus survey

## Abstract

Procellariiformes are among the most threatened seabirds globally, yet viral pathogens affecting this group remain poorly understood. Herpesviruses are widespread in birds and capable of establishing latency, reactivating under immunosuppression, and causing severe disease, particularly in cross-species transmission. We surveyed herpesvirus in 50 individuals (12 species) that stranded along the southeastern Brazilian coast (2017–2023). A nested pan-PCR protocol targeting the DNA polymerase gene detected herpesvirus DNA in 24% (12/50) of the birds. Seven distinct herpesvirus sequence types were identified, all clustering within the genus *Mardivirus*, including two previously known variants and novel lineages. To our best knowledge, this represents the first herpesvirus report in the Black-browed Albatross (*Thalassarche melanophris*), Cape Verde Shearwater (*Calonectris edwardsii*), Manx Shearwater (*Puffinus puffinus*), and Southern Giant-Petrel (*Macronectes giganteus*), as well as new host reports for the Yellow-nosed Albatross (*T. chlororhynchos*) and Cory’s Shearwater (*C. borealis*). No associated lesions were observed in histopathology. Our findings expand the known host range of herpesviruses in Procellariiformes and reveal possible circulation among colonies and non-breeding areas across the Southern and Northern hemispheres. Our results underscore the need for integrative ecological-, virological-, and population-based studies to clarify how seabird ecology influences herpesvirus transmission dynamics and long-term persistence in this group.

## Simple Summary

Albatrosses, petrels, and shearwaters are among the most threatened group of birds worldwide. The known threats faced by these seabirds include human-related factors such as entanglement in fishing lines and climate change; however, studies on other potential causes, such as infectious agents, are scarce. Herpesviruses can cause lifelong infection without clinical signs, but can reactivate under immunosuppression and cause severe disease and/or mortality. We surveyed herpesviruses in 50 individuals (12 species) that stranded along the southeastern Brazilian coast between 2017 and 2023. We detected herpesviruses in 24% (12/50) of the birds. This study described the first herpesvirus report in the Black-browed Albatross (*Thalassarche melanophris*), Cape Verde Shearwater (*Calonectris edwardsii*), Manx Shearwater (*Puffinus puffinus*), and Southern Giant-Petrel (*Macronectes giganteus*), and confirmed the Yellow-nosed Albatross (*T. chlororhynchos*) and Cory’s Shearwater (*C. borealis*) as species that can be infected with these viruses, as previously reported. No lesions compatible with herpesvirus-caused disease were observed. Our findings contribute to the current knowledge on herpesviruses in albatrosses, petrels, and shearwaters and show that herpesviruses are present in species with different geographic ranges and breeding colonies, highlighting the need to clarify transmission dynamics and persistence of herpesviruses in this avian group.

## 1. Introduction

Procellariiformes, comprising albatrosses (Diomedeidae), petrels and shearwaters (Procellariidae), southern storm-petrels (Oceanitidae), and northern storm-petrels (Hydrobatidae), are among the most threatened avian orders worldwide [1,2]. These birds are genuine marine species, with a global distribution, and are particularly abundant in the Southern Hemisphere [3]. Procellariiformes fulfill critical ecological functions within marine ecosystems and are crucial to biodiversity, including biomass consumption and nutrient regulation and transfer to marine environments [4,5,6]. Thus, Procellariiformes are considered key bioindicators of ocean health, capable of reflecting environmental changes, such as fluctuations in fish stocks, plastic pollution, shifts in trophic dynamics, and dispersion of contaminants [6,7,8,9,10]. Along the Brazilian coast, at least 45 Procellariiformes species are known to be either breeding residents, vagrants, or non-breeding visitors, including several threatened species [11].

The current knowledge regarding the threats faced by Procellariiformes is heterogeneous, mainly focused on anthropogenically related (e.g., bycatch, impact of invasive species in breeding areas, pollution) and environmental conditions [2,12]. In contrast, the role of infectious agents in the population dynamics of this group remains poorly understood, partly because their effects are often sublethal or overlooked. Studies on viral infectious agents in Procellariiformes were limited to avipoxviruses, influenza A virus, and herpesvirus in albatrosses, shearwaters, and petrels [13,14,15]. More recently, additional viral taxa, including astrovirus, calicivirus, flavivirus, papillomavirus, picornavirus, and rotavirus, have been identified in some shearwaters and a prion species [16]. Although infectious diseases are recognized as an important driver in population ecology, as seen in the highly pathogenic avian influenza A (HPAI) H5N1 2.3.3.4b subtype outbreak [17], evaluating their conservation impact is challenging. This is particularly true in Procellariiformes, due to their pelagic life story and scarce studies on pathogens that may cause chronic and sublethal effects in their populations, as well as the limited knowledge about the epidemiology of the infectious agents affecting this avian order [18].

Herpesviruses are large, linear, double-stranded DNA viruses of the family *Orthoherpesviridae*, classified into three subfamilies: *Alpha*-, *Beta-* and *Gammaherpesvirinae* [19]. To date, only alphaherpesviruses have been reported in birds [19], specifically from the genera *Iltovirus* and *Mardivirus*. These viruses establish lifelong latent infections in their natural hosts [13,20], can reactivate under immunosuppression, and may cause severe disease or mortality [21], especially following cross-species transmission [22]. Avian herpesviruses are typically transmitted through oral, respiratory, and cloacal secretions [23]. Clinical signs of infection in wild birds are usually nonspecific, including depression, conjunctivitis, reduced/absent appetite, regurgitation, biliverdinuria, diarrhea, or sudden death [24]. In poultry, doves, and psittacines, herpesviruses can cause diseases of economical relevance, such as the infectious laryngotracheitis virus, Marek’s disease virus, Pacheco’s parrot disease virus, duck plague virus/duck viral enteritis, and the one caused by *Columbid alphaherpesvirus 1* [21,24,25,26,27].

In seabirds, herpesviruses have been detected in multiple avian orders, including terns and gulls (Charadriiformes), loons (Gaviiformes), tropicbirds (Phaethontiformes), penguins (Sphenisciformes), frigatebirds and boobies (Suliformes), and shearwaters and albatrosses (Procellariiformes) [13,16,28,29,30,31,32,33]. Although clinical manifestations of infection in wild birds are often nonspecific, herpesviruses can be pathogenic in seabirds. For instance, Magellanic penguin herpesvirus 1 has been implicated in a mortality outbreak in Magellanic Penguins (*Spheniscus magellanicus*) under rehabilitation in Brazil, characterized by necrotic-hemorrhagic tracheitis [13]. Similarly, recurrent large-scale mortality events observed in French Guiana populations of Magnificent Frigatebird (*Fregata magnificens*) have been attributed to Fregata magnificens herpesvirus 1 [34,35], which may be associated with immunosuppression, malnutrition, and environmental pollution [36,37]. To date, herpesviruses have been reported in only four Procellariiformes species: Yellow-nosed Albatross (*Thalassarche chlororhynchos*, family Diomedeidae), Cory’s Shearwater (*Calonectris borealis*, fam. Procellariidae), and Great Shearwater (*Ardenna gravis*, Procellariidae) of Brazil, and in Sooty Shearwater (*Ardenna grisea*, Procellariidae) of New Zealand, all without associated lesions [13,16,33].

Due to the limited knowledge regarding herpesviruses in Procellariiformes, the goal of this study was to survey and identify herpesviruses in wild Procellariiformes stranded along the southeastern coast of Brazil.

## 2. Materials and Methods

### 2.1. Samples

We analyzed 50 birds of 12 Procellariiformes species: (i) Diomedeidae—Yellow-nosed Albatross (n = 1) and Black-browed Albatross (*Thalassarche melanophris*, n = 8); (ii) Oceanitidae—Wilson’s Storm-Petrel (*Oceanites oceanicus*, n = 5); and (iii) Procellaridae—Great Shearwater (n = 4), Cory’s Shearwater (n = 9), Cape Verde Shearwater (*Calonectris edwardsii*, n = 1), Cape Petrel (*Daption capense*, n = 1), Southern Giant-Petrel (*Macronectes giganteus*, n = 4), Antarctic Prion (*Pachyptila desolata*, n = 2), White-chinned Petrel (*Procellaria aequinoctialis*, n = 7), Atlantic Petrel (*Pterodroma incerta*, n = 2), and the Manx Shearwater (*Puffinus puffinus*, n = 6) (Table 1).

**Figure 1:**
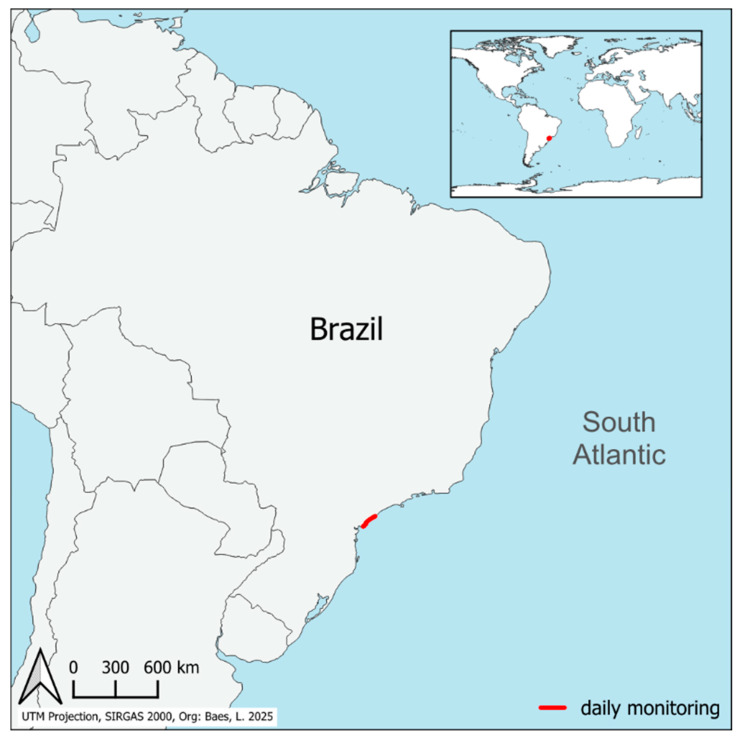
Coastline monitored daily by the Instituto de Pesquisas Cananéia (IPeC), southeastern Brazil, where the Procellariiformes studied herein stranded.

All birds were necropsied following standard procedures [38,39] by the Instituto de Pesquisas de Cananéia (IPeC) between 2017 and 2023. The IPeC is responsible for the daily monitoring of 120.94 km of the southern coast of São Paulo state (Cananéia—SP, 25°0′54″ S 47°55′37″ W), Brazil (Figure 1). Most animals stranded and were rescued alive, dying later on while under care (n = 27), while the remaining individuals stranded dead (n = 23). Sex and age determinations were based on visualization of the gonads and feather pattern during necropsy, respectively [40]. Body condition index was scored according to a four-point scale [41], divided into 4 categories: cachectic, poor, moderate, and good. Major breeding of each species was classified into the oceans Atlantic, Indian, Pacific, and/or Antarctic, according to BirdLife Datazone [42]. All selected carcasses presented a score of 2, in fresh condition [43]. Brain, intestines, kidneys, liver, lungs, and spleen were collected in duplicate and frozen at −80 °C for molecular testing and stored in 10% buffered formalin for histopathology.

### 2.2. Molecular Techniques

Total DNA was extracted from selected tissue samples—lungs (n = 50), kidneys (n = 50), and brain (n = 47) using the PureLink Viral RNA/DNA Mini Kit (Invitrogen, Waltham, Massachusetts, USA), following the manufacturer’s instructions. DNA extractions were tested for herpesviruses with a nested PCR that amplifies a 215–315 bp fragment of the DNA polymerase (DPOL) gene [44]. In herpesvirus-positive cases (HV-positive), available intestines (n = 5), liver (n = 11), and spleen (n = 9) samples were also tested. Adequate positive and no-template controls were added to all reactions. Amplicons of the expected size were purified with ExoSap-IT (USB Corporation, OH, USA) and directly sequenced in both directions. The quality of the obtained sequences was visually assessed, and the consensus sequences were constructed based on the alignment of the forward and reverse sequences performed on Mega 11 [45]. The consensus sequences were compared by BLAST search to those kept on the GenBank/EMBL/DDBJ database (https://blast.ncbi.nlm.nih.gov/Blast.cgi, accessed on 10 September 2025).

### 2.3. Histopathology

Light microscopy histopathological evaluation was performed in 5 μm thick formalin-fixed paraffin-embedded tissue sections, stained with hematoxylin and eosin, of all the available tissues from the herpesvirus-PCR-positive individuals

### 2.4. Statistical Analysis

To evaluate whether the epidemiology of herpesvirus in Procellariiformes is influenced by sex (female, male) and age class (juvenile, adult), a quasi-binomial generalized linear model (GLM) was fitted to data of HV-positive animals. The significance of the effects was assessed using F tests. Goodness-of-fit was assessed using “half-normal plot” graphs with simulated envelopes at the 95% level [46].

## 3. Results

### 3.1. Molecular Findings

Overall, the herpesvirus prevalence was 24% (12/50) (Table 2), comprising six HV-positive species: Yellow-nosed Albatross (100%, 1/1), Cape Verde Shearwater (100%, 1/1), Black-browed Albatross (88%, 7/8), Southern Giant-Petrel (25%, 1/4), Manx Shearwater (17%, 1/6), and Cory’s Shearwater (11%, 1/9). The remaining species were herpesvirus-negative. Of the herpesvirus-positive cases, seven were females and five were males, and six were juveniles and another six were adults. Herpesvirus infection was found in individuals with either cachectic (n = 7) or poor (n = 5) body condition. According to the targeted tissue sample, we observed apparently higher prevalence in lungs (20%, 10/50), followed by kidneys (16%, 8/50) and brain (11%, 5/47), and among the remaining tested tissues of PCR-positive animals, in intestines (80%, 4/5), spleen (78%, 7/9), and liver (64%, 7/11).

**Figure 2:**
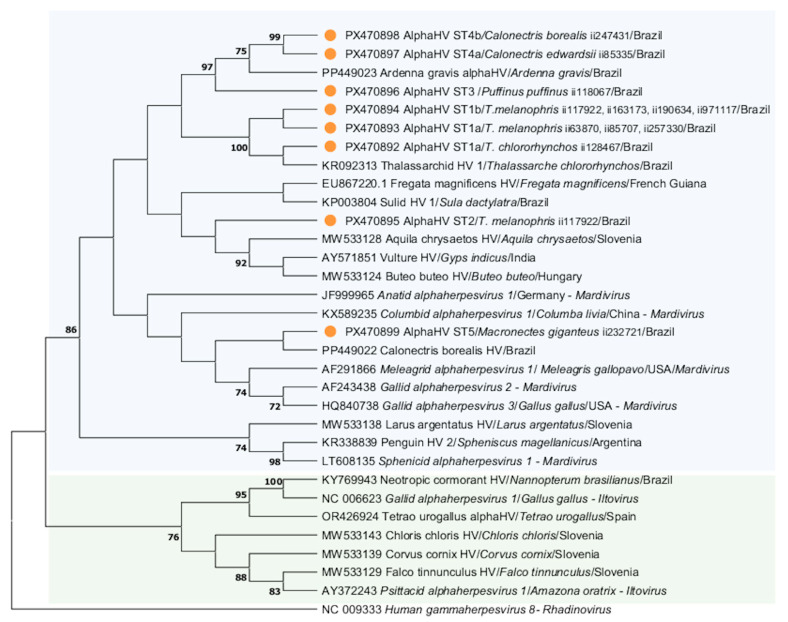
Maximum likelihood phylogenetic tree based on the Jones–Taylor–Thornton model with invariant sites and gamma distributed of the deduced amino acid herpesviral sequences: (i) obtained in Procellariiformes in this study (orange dots, labeled with case numbers), (ii) representative alphaherpesvirus species (in italics and written in full) within the genera *Mardivirus* and *Iltovirus*, (iii) *Human gammaherpesvirus* 8 as outgroup. Bootstrap values below 70 are not shown. The analysis was performed with 1000 bootstrap replications. The blue and the green squares comprised herpesvirus sequences that clustered within *Mardivirus* and *Iltovirus* genera, respectively. HV = herpesvirus.

Overall, seven herpesvirus sequence types (STs) were found: ST1a in three Black-browed Albatross (case numbers ii63870, ii85707, and ii257330) and one Yellow-nosed Albatross (ii128467), ST1b in four Black-browed Albatross (ii117922, ii163173, ii190634, and ii971117), ST2 in a Black-browed Albatross (ii117922), ST3 in a Manx Shearwater (ii118067), ST4a in a Cape Verde Shearwater (ii85335), ST4b in a Cory’s Shearwater (ii247431), and ST5 in a Southern Giant-Petrel (ii232721). One Black-browed Albatross (ii117922) was co-infected by two STs (i.e., ST1b and ST2). Representative consensus sequences corresponding to the STs described above were submitted to GenBank under accession numbers PX470892-PX470899. When more than one host species was associated with the same ST, sequences from both hosts were deposited (i.e., PX470892 and PX470893, found in Black-browed and Yellow-nosed Albatrosses, respectively). According to the phylogram (Figure 2), all the alphaherpesvirus sequences found in the studied seabirds clustered into the *Mardivirus* genus.

ST1a and ST1b differed by only a single non-coding mutation, with nucleotide (nt) and amino acid (aa) identities of 99.4% and 100% between them, respectively. ST4a and ST4b differed by two non-coding mutations, with nt and aa identities of 98.8 and 100%, respectively. In comparison to previous known *Mardivirus* genus in other seabirds, (i) ST1a and ST1b presented nt identities of 100% and 99.4%, respectively, with an alphaherpesvirus described in a Yellow-nosed Albatross of Brazil (KR092313); (ii) ST2 had nt and aa identities of 65.7% and 67.3%, respectively, to an alphaherpesvirus sequence (EU867220) found in a Magnificent Frigatebird of French Guiana; (iii) ST3 presented nt and aa identities of 83.7% and 85.5%, respectively, to an alphaherpesvirus found in Great Shearwaters (*Ardenna gravis*, PP449023) of Brazil; (iv) ST4a and ST4b had nt and aa identities of 88.6% and 89.1%, respectively, to an alphaherpesvirus found in Great Shearwaters (PP449023); and (v) ST5 had nt and aa identities of 64.2% and 63.6%, respectively, to Columbid herpesvirus 1 (e.g., KJ995972, MW625922; KX589235) of domestic pigeons (*Columba livia*).

### 3.2. Gross and Histopathological Findings

Overall, the HV-positive individuals presented lesions associated with starvation, septic shock, trauma, and respiratory system conditions (i.e., pneumonia and respiratory failure). One individual—a Black-browed Albatross (ID 971117) presented lymphoplasmacytic periganglioneuritis, which could potentially be associated with herpesvirus infection. Nevertheless, such association could not be confirmed due to the absence of inclusion bodies. No histopathology lesions characteristic of herpesvirus infection were observed in this study. Gross and histopathological findings are presented in detail in the Supplementary Material (Table S1).

### 3.3. Relationship Between Sex and Age, and Herpesvirus Infection

The occurrence of herpesvirus infection was not significantly associated with either the sex (F_1_,_1_ = 1.068, *p* = 0.311) or age class (F_1_,_1_ = 0.434, *p* = 0.516) of the individuals.

## 4. Discussion

To the authors’ knowledge, this is the most extensive herpesvirus survey conducted in Procellariiformes to date, providing the first evidence of herpesvirus infection in four species within this order: Black-browed Albatross, Cape Verde Shearwater, Manx Shearwater, and Southern Giant-Petrel. Additionally, we confirm herpesvirus infection in two other species in which these viruses had been previously detected—Yellow-nosed Albatross (one individual) [13] and Cory’s Shearwater (two individuals) [33]. All herpesviruses identified in this study belong to the genus *Mardivirus*, which primarily infects birds, including both domestic and wild species. Some members of this genus, such as *Gallid alphaherpesvirus 2*, are highly pathogenic and cause Marek’s disease [47]. No HV-related histopathology lesions were observed in the positive individuals, possibly because they did not show clinical signs at time of death. Herein, the detection of *Mardivirus* across multiple Procellariiformes suggests potential epidemiological and conservation implications, raising important questions about the role of such viruses in seabird health and population dynamics [33].

The herpesvirus sequence types found in three Black-browed Albatrosses (ST1a), one Yellow-nosed Albatross (ST1a), and in four Black-browed Albatrosses (ST1b) were identical at the amino acid level to the sequence previously reported by [13] in a Yellow-nosed Albatross stranded in southern Brazil and could have a common origin. Although these albatross species breed in geographically distinct locations of the Southern Hemisphere—the majority of Black-browed Albatrosses on the Malvinas Islands and most Yellow-nosed Albatrosses on Tristan da Cunha and Gough Island—both share non-breeding areas along the Southern Atlantic during the austral winter [48]. The presence of nearly identical viral sequences across two *Thalassarche* species [49] may reflect long-term host–virus association, through the infection of early *Thalassarche* lineages by an ancestral herpesvirus, as observed in herpesvirus infections in species like West Indian (*Trichechus manatus*) and Amazonian manatees (*Trichechus inunguis*) [50], given that herpesviruses typically co-diverge with their hosts [51]. Alternatively, it may be explained by interspecies transmission, since the two species share overlapping foraging areas, which provides opportunities for viral exchange. Nevertheless, differences in ecological niches, such as feeding strategy [52,53], may limit opportunities for viral spillover. The repeated detection of highly similar viral variants in multiple individuals of two closely related hosts suggests that a species within *Thalassarche* genus likely represents the natural host. Our findings confirm that this virus has circulated in albatrosses of the Southern Atlantic since its first identification in stranded animals in 2013 [13], with environmental circulation into different breeding areas.

The sequence types found in one Black-browed Albatross (ST2), one Manx shearwater (ST3), one Cape Verde shearwater (ST4a), and one Southern Giant-Petrel (ST5) likely correspond to novel alphaherpesvirus species, based on (i) the low identity of a well-preserved gene with the closest ones from GenBank/DDBJ/ENA observed on the identity analyses and (ii) the identification in a novel host species. The ST2 found in an albatross of the genus *Thalassarche* clustered distantly in the phylogenetic tree from ST1 herpesvirus found in birds of the same genus, which may indicate a distinct virus evolutionary trajectory. ST3, ST4a, and ST4b herpesvirus sequences—found in Manx Shearwater, a Cape Verde Shearwater and a Cory’s Shearwater, respectively—presented amino acid similarities ranging from 85.5% to 89.1% to a herpesvirus found in Great Shearwaters stranded in Brazil [33] and are classified into the same cluster in our phylogenetic analyses. All of them infect shearwater species of the Atlantic Ocean. The Manx, Cape Verde, and Cory’s Shearwaters are breeders from the Northern Hemisphere, while Great Shearwaters breed in subantarctic and temperate islands of the Southern Hemisphere [48].

The ST identified in a Cory’s Shearwater (ST4b) was very similar to the one found in a Cape Verde Shearwater (ST4a) and could correspond to a variant and potentially have a common origin. Of note, that sequence was not similar to herpesviruses previously reported in two Cory’s Shearwaters stranded in northeastern Brazil [33] and represents the second herpesvirus described in that species. Both *Calonectris* species likely diverged during the Pleistocene through allopatric speciation, with the Cape Verde shearwater deriving from the Mediterranean subspecies (Scopoli’s Shearwater, *Calonectris diomedea*) and establishing an endemic population in the Cape Verde Archipelago, while the Cory’s Shearwater established colonies in the Northeastern Atlantic [54]. This evolutionary history may suggest a long-term host–virus association, as our findings reveal herpesvirus sequences distinct from those previously reported in Cory’s Shearwater [33], indicating that at least two herpesviruses could be circulating within the genus and one that may be circulating into two different shearwater species. Differently from previous mentioned Thalassarche species, the Cape Verde, Cory’s, and Scopoli’s Shearwaters are closely related pelagic seabirds, sharing non-breeding distributions in the Atlantic [55], which could facilitate viral transmission. Although Scopoli’s Shearwater was not assessed in this study, further surveys are needed to clarify herpesvirus diversity and co-evolution within this genus, particularly given the potential origin of the Cape Verde Shearwater from the Mediterranean lineage and a non-similar herpesvirus previously reported in two Cory’s Shearwaters.

Furthermore, we found a distinct herpesvirus sequence type in a Southern Giant-Petrel (ST5). The sequence obtained was highly divergent (>35%) when compared to the closest one from public databases, likely corresponding to a specific virus evolutionary trajectory in this species. Southern Giant-Petrels are highly mobile through the Southern Hemisphere, traveling across vast oceanic areas and visiting multiple colonies [56,57]. Therefore, herpesvirus may circulate among a broader range of populations beyond the South Atlantic, although surveys from other ocean locations are still lacking.

The absence of significant differences in herpesvirus infection rates between sexes or among age classes suggests that intrinsic host characteristics may not be major determinants of susceptibility [24]. Nonetheless, intraspecific sexual differences in foraging behavior, such as longer or more distant trips and divergent feeding strategies [58,59,60,61,62], could increase the likelihood of pathogen exposure in one sex in comparison to the other. Even if one group is more frequently exposed, the virus could still spread effectively among other individuals through shared breeding sites and social interactions. All infected individuals in this study were in cachectic or poor body condition, which is a common condition among Procellariiformes stranded in Brazil [63,64]. Poor nutritional conditions compromise immune function [65] and can trigger reactivation of latent viruses and the development of associated lesions, as previously observed in other seabirds [31,36]. However, no associated lesions were observed in the individuals examined in this study.

Despite the extensive survey conducted, certain limitations should be noted. First, the relatively short length of the sequences obtained allows only a limited resolution of phylogenetic relationships among the herpesviruses. Second, some species were represented by only one or a few individuals, which may influence the assessment of viral prevalence and diversity. Third, our study is geographically limited to the southeastern coast of Brazil, and further surveys across other oceanic regions are needed to better understand the broader distribution of these viruses.

## 5. Conclusions

This study expands the known herpesvirus host range in Procellariiformes, providing the first evidence of infection in several species, including the first report in petrels. The phylogenetic patterns indicate herpesvirus co-evolution with Atlantic Procellariiformes, host-associated clustering in shearwaters and albatrosses, and circulation among colonies and non-breeding areas across both hemispheres. Our findings show that, despite geographically distinct colonies and divergent ecological niches, certain herpesviruses could infect multiple seabird species. Future studies on ecology, pathology, and virology (e.g., viral surveillance in breeding colonies, complete sequencing) are required to clarify how seabird ecology drives herpesvirus transmission and to assess the impact of these viruses on Procellariiformes.

## Figures and Tables

**Table 1:** Family, species, conservation status according to the International Union for Conservation of Nature (IUCN), major breeding sites, number of individuals (n), sex, age class, and body condition of the Procellariiformes tested for herpesvirus in this study.

Family	Species	IUCN Red List *	Major Breeding Sites **	n	Sex ***		Age ***		Body Condition			
					F	M	J	A	Cachectic	Poor	Moderate	Good
Diomedeidae	Yellow-nosed Albatross (*Thalassarche chlororhynchos*)	EN	A (S)	1	1	0	0	1	1	0	0	0
	Black-browed Albatross (*Thalassarche melanophris*)	LC	A, I, P (S)	8	4	4	5	3	2	6	0	0
Oceanitidae	Wilson’s Storm-Petrel (*Oceanites oceanicus*)	LC	A, I (S)	5	2	3	1	2	2	2	1	0
Procellariidae	Great Shearwater (*Ardenna gravis*)	LC	A (S)	4	2	2	3	1	4	0	0	0
	Cory’s Shearwater (*Calonectris borealis*)	LC	A (N)	9	3	6	4	4	8	0	0	1
	Cape Verde Shearwater (*Calonectris edwardsii*)	NT	A (N)	1	0	1	1	0	1	0	0	0
	Cape Petrel (*Daption capense*)	LC	Antarctic	1	0	1	0	1	0	0	1	0
	Southern Giant-Petrel (*Macronectes giganteus*)	LC	A, I (S)	4	3	1	1	3	3	1	0	0
	White-chinned Petrel (*Procellaria aequinoctialis*)	VU	A, I, P (S)	7	5	2	5	2	3	2	0	2
	Antarctic Prion (*Pachyptila desolata*)	LC	A, I, P (S)	2	1	1	0	1	2	0	0	0
	Atlantic Petrel (*Pterodroma incerta*)	EN	A (S)	2	1	1	1	0	1	1	0	0
	Manx Shearwater (*Puffinus puffinus*)	LC	A (N)	6	2	4	2	4	3	0	3	0
**Overall**				**50**	**24**	**26**	**23**	**22**	**30**	**12**	**5**	**3**

* LC = least concern, NT = near-threatened, VU = vulnerable, EN = endangered; ** A = Atlantic Ocean: South (S) and North (N), I = Indian Ocean: South (S), P = Pacific Ocean: South (S) and North (N); *** F = female, M = male, J = juvenile, A = adult.

**Table 2:** Prevalence of herpesvirus in Procellariiformes detected in this study and biological characteristics of the herpesvirus-positive birds.

Family	Species	Herpesvirus Prevalence *	Biological Characteristics of Herpesvirus-Positive Birds							
			Sex		Age		Body Condition			
			F	M	J	A	Cachectic	Poor	Moderate	Good
Diomedeidae	Yellow-nosed Albatross (*Thalassarche chlororhynchos*)	100% (1/1)	1	-	-	1	1	-	-	-
	Black-browed Albatross (*Thalassarche melanophris*)	88% (7/8)	4	3	4	3	2	5	-	-
Procellariidae	Cory’s Shearwater (*Calonectris borealis*)	11% (1/9)	-	1	-	1	1	-	-	-
	Cape Verde Shearwater (*Calonectris edwardsii*)	100% (1/1)	-	1	1	-	1	-	-	-
	Southern Giant-Petrel (*Macronectes giganteus*)	25% (1/4)	1	-	-	1	1	-	-	-
	Manx Shearwater (*Puffinus puffinus*)	17% (1/16)	1	-	1	-	1	-	-	-
**Overall**		**24% (12/50)**	**7**	**5**	**6**	**6**	**7**	**5**	**0**	**0**

* Number of HV-positive/Total number of tested individuals pertaining to this species in this study.

## Data Availability

All data generated or analyzed during this research are included in the manuscript. The nucleotide sequences obtained in this research were submitted to GenBank under accession numbers PX470892-PX470899.

## Supplementary Materials

The following supporting information can be downloaded at https://www.mdpi.com/article/10.3390/vetsci12121143/s1. Table S1: Individual identification (ID), species, sex, age, body condition, gross findings, and histopathology of the Procellariiformes analyzed in this study.
