# Supplementary material for: Herpesvirus Diversity in Atlantic Procellariiformes
Source: Vet Sci. 2025 Nov 29;12(12):1143. doi: 10.3390/vetsci12121143 (PMC12737773; doi:10.3390/vetsci12121143)
Supplement: Supplementary file 1 [file vetsci-12-01143-s001.zip › vetsci-3983089-supplementary.pdf]

Supplementary Table S1. Individual identification (ID), species, sex, age, body condition, gross findings, and histopathology of the Procellariiformes analyzed in this study.

ID	Species	Sex	Age class	Body condition	Gross Findings	Histopathology
128467	Yellow-nosed Albatross (*T. chlororhynchos*)	F	A	cachectic	Heart atrophy with moderately engorged vessels. Pectoral muscle atrophy. Pulmonary edema. Moderate and diffuse renal parenchyma congestion.	Rare renal tubules filled with discrete amount of trematode eggs, some surrounded by lymphohistiocytic inflammatory infiltrate. No alterations were found in adrenal gland, spleen, brain, heart, stomach, liver, spinal cord, muscle, lungs, esophagus, trachea, and thyroid. Autolysis prevented analysis of intestines.
971117	Black-browed Albatross (*T. melanophris*)	M	J	cachectic	Atrophied cardiac fat deposits. Generalized congestion. Multifocal whitish caseous material in the proximal third of the mucosal esophagus.<br>Ovoid tracheal mass (approx. 2 cm) with adjacent yellowish cystic nodules containing caseous debris. Focal and mild deposition of caseous material in the right cranial abdominal air sac.<br>Diffuse moderate pulmonary congestion and edema. Thickened air sacs with diffuse deposition of yellowish granules.	Lymphoplasmacytic periganglioneuritis, mild and focally extensive. Esophagus with perivascular lymphoplasmacytic and heterophilic inflammatory infiltrate in lamina propria. Liver with macrovacuolar hepatic degeneration, multifocal and mild to moderate, and islands of extramedullary hematopoiesis, multifocal, mild. Small intestine with necrotizing heterophilic enteritis, multifocal, moderate, acute, associated with intralesional bacteria, and lymphoplasmacytic enteritis, diffuse, moderate, chronic. Granulomatous and heterophilic tracheitis, focally extensive, subacute, severe, associated with presence of coccobacillary bacteria. Ulcerative granulocytic dermatitis, focally extensive and severe with intralesional bacteria. Mild proteinuria in renal tubules with presence of microgamonts and macrogamonts of coccidia within tubules. No alterations were found in spleen, brain, heart, pancreas, lung, air sacs, and thyroid.
117922	Black-browed Albatross (*T. melanophris*)	M	A	poor	Diffuse and moderate brain congestion. Diffuse and moderate heart congestion, with mild cardiac fat atrophy. Pulmonary congestion and presence of brownish viscous liquid content near the bronchial insertion suggestive of aspiration. The same liquid was present in the trachea. Renal congestion. Diffuse and moderate congested adrenal gland. Proventriculus presenting mild multifocal yellowish foci in mucosa. Liver presenting friable parenchyma. Intestine mucosa presenting multifocal congestion. Presence of whitish fibrous periarticular tissue in the interphalangeal joint of the right hind limb, thickened and yellowish tendon, and total loss of movement between phalanges of fourth digit. Left hind limb with mild abrasions and healed	Congestion in adrenal gland. Mild hemosiderosis in spleen. Mild congestion in brain and heart. Perivascular lymphocytic and histiocytic inflammatory infiltrate in esophagus. Mild diffuse chronic lymphoplasmacytic proventriculitis. Diffuse chronic lymphoplasmacytic ventriculitis, with focus of calcification in mucosa, presence of yeasts with rare pseudohyphae. Mild diffuse lymphoplasmacytic enteritis and autolysis in small intestine. Multifocal to coalescing moderate necrotizing granulomatous myositis, with hemorrhage, edema, and hemosiderosis in pectoral musculature. Focally extensive moderate acute heterophilic ulcerative dermatitis with coccoid bacteria and hemosiderosis. Air sacs with moderate congestion

					perforation in the interdigital membrane, swelling on plantar surface of the fourth digit interphalangeal joint.	and presence of yellowish refractile material. Lung with hyaline, eosinophilic, and amorphous substance, myriads of bacilli, amorphous yellow refractile material and some epithelial cells filling the lumen of parabronchi, multifocal and markedly; their blood vessels are diffusely filled and dilated by erythrocytes; moderate congestion and moderate heterophilia in capillaries. Kidneys with moderate chronic focally extensive lymphoplasmacytic ureteritis. No alterations were found in gonads, large intestine, and thyroid.
190634	Black-browed Albatross (*T. melanophris*)	M	J	cachectic	Moderate to severe focal subarachnoid hemorrhage. Generalized congestion. Cardiac atrophy. Moderate to severe pulmonary edema. Renal congestion. Presence of moderate multifocal to coalescing miliary nodules and parasites in the mucosa of the esophagus and stomach. Atrophied pectoral musculature. Presence of yellowish caseous granulomes (approx. 2 cm) with skin disruption in ventral portion of neck.	Spleen with mild multifocal hemosiderosis and diffuse mild histiocytosis. Ulcerative granulocytic and histiocytic esophagitis, multifocal and moderate associated with intralesional bacteria. Ulcerative granulocytic and histiocytic proventriculitis, multifocal and moderate associated with intralesional bacteria. Liver with mild multifocal periportal lymphocytic inflammatory infiltrate. Muscle with mild myocyte atrophy. Ulcerative granulocytic and histiocytic dermatitis, focally extensive and moderate associated with intralesional bacteria. Lung with diffuse moderate congestion. Kidney with presence of protozoa in collecting tubules, associated with multifocal moderate lymphohistiocytic inflammatory infiltrate. No alterations were found in adrenal gland, heart, and thyroid. Autolysis prevented analysis of intestines.

257330	Black-browed Albatross (*T. melanophris*)	F	J	poor	Atrophy of cardiac fat. Ulcerative lesions in the mucosa and caseous adherence in the middle third of the esophagus, suggestive of esophageal perforation.<br><br>Severe intestinal parasitism.<br><br>Congested intestinal mucosa and cloaca.<br>Subcutaneous caseous granuloma present on the cervical region, adhered to skin, musculature, esophagus and cervico-cephalic air. Multifocal and moderate greenish plaques in the bronchial insertion. Enlarged right lung with bulging borders enlarged in comparison to the left lung. Marbled kidneys. Thickened and congested clavicular air sac presenting diffuse and severe caseous material.	Bursa with marked lymphoid depletion. Brain and cerebellum with congestion, multifocal and mild to moderate. Transmural granulocytic and granulomatous esophagitis, focally extensive and severe associated with intralesional bacteria. Erosive granulocytic and histiocytic proventriculitis, multifocal and severe associated with intralesional bacteria. Liver with diffuse moderate macrovacuolar degeneration, hypertrophy, and hyperplasia of Kupffer cells with mild intracytoplasmic, granular, brownish pigmentation. Small intestine with mild diffuse lymphoplasmacytic enteritis, with foci of mineralization. Diffuse severe granulocytic and histiocytic dermatitis and panniculitis associated with intralesional bacteria. Lung with diffuse severe granulocytic and granulomatous pneumonia associated with intralesional bacteria, with foci of hemorrhage and congestion. Kidneys with focus of mineralization, presence of protozoa associated with multifocal mild histiocytic inflammatory infiltrate in collecting tubules. Air sacs with diffuse severe granulocytic and granulomatous airsacculitis associated with intralesional bacteria. No alterations were found in adrenal gland, heart, large intestine, pectoral muscle, gonads, and trachea.
85707	Black-browed Albatross (*T. melanophris*)	F	A	poor	Cardiac congestion. Focal and mild to moderate caseous material adhered to the ventricular mucosa. Mandible fracture and associated severe caseous inflammatory process. Lung congestion, edema, and pneumonia.	Moderate multifocal heterophilic and histiocytic pneumonia associated with bacterial colonies, mild congestion, and moderate autolysis. Kidney with mild amount of bacteria and cellular debris inside collecting tubules. Autolysis prevented analysis of spleen, heart, stomach, liver, muscle, bones, and thyroid.
63870	Black-browed Albatross (*T. melanophris*)	F	J	poor	Generalized organ congestion. Multifocal to coalescing, mild to moderate whitish spots on the epicardium. Moderate edema and pulmonary congestion. Multifocal and mild whitish granulomas in the anterior left thoracic air sac. Multifocal and moderate esophageal mucosal cysts of approximately 0.5 cm in diameter.	Spleen with mild heterophilia and diffuse histiocytosis. Liver with diffuse microvacuolar degeneration of hepatocytes. Small intestine with diffuse moderate chronic lymphohistiocytic enteritis. Lung with multifocal moderate hemorrhage in parabronchi. Stomach with diffuse mild heterophilic and lymphoplasmacytic ventriculitis. No alterations were found in adrenal gland, brain and cerebellum, heart, spinal cord, pectoral muscle, kidney, thyroid, and trachea.

163173	Black-browed Albatross (*T. melanophris*)	F	A	poor	Generalized congestion. Hydropericardium. Moderate to severe pulmonary congestion and edema. Ventriculum presenting remaining food contents and mucosal ulcers of approx. 1 cm in diameter associated with caseous material and blood.	Stomach with focally extensive moderate heterophilic and histiocytic ventriculitis, with intralesional bacteria. Moderate congestion in lungs. Liver with mild multifocal periportal lymphoplasmacytic inflammatory infiltrate. Kidneys with mild congestion. No alterations were found in muscle, spinal cord, skin, heart, and brain. Autolysis prevented analysis of pancreas and lung.
247431	Cory's Shearwater (*C. borealis*)	M	A	cachectic	Pulmonary congestion. Congested liver. Atrophied pectoral musculature.	Proventriculus with mild perivascular lymphoplasmacytic inflammatory infiltrate in submucosa and multiple rod-shaped bacteria in lumen. Small and large intestine with multifocal mild lymphoplasmacytic enteritis, and multiple rod-shaped bacteria in lumen. Muscle with mild atrophy. Lung with mild congestion. Kidneys with ectasia of collecting ducts associated with presence of adult trematode parasite, minimal epithelial necrosis. No alterations were found in adrenal gland, brain, heart, ventriculus, gonads, and skin.
85335	Cape Verde Shearwater (*C. edwardsii*)	M	J	cachectic	Atrophied pectoral musculature.<br><br>Bilateral interdigital yellowish nodules in the plantar region. Pulmonary congestion and edema. Presence of sanguineous fluid in trachea and oral cavity.	Liver with mild multifocal macrovacuolar degeneration of hepatocytes, mild periportal lymphocytic inflammatory infiltrate, and mild hypertrophy of Kupffer cells with intracytoplasmic, brownish, granular pigmentation. Gonads with focal focus of mineralization in medullary tubules and mild focal lymphocytic inflammatory infiltrate in collecting tubules. Trachea with mild focal lymphocytic inflammatory infiltrate in lamina propria. No alterations were found in muscle, lung, and thyroid.
232721	Southern Giant-Petrel (*M. giganteus*)	F	A	cachectic	Generalized congestion. Moderate hydropericardium. Moderate diffuse multifocal red spots on the liver. Atrophied pectoral musculature. Perforations (0.2 to 2 cm) on interdigital membranes. Mild to moderate whitish friable material over the right abdominal air sac surface. Pulmonary edema. Focal tracheal congestion (adjacent to the syrinx).	Adrenal gland with moderate congestion. Spleen with mild diffuse heterophilic splenitis. Brain with moderate congestion. Liver with moderate hepatocyte atrophy, multifocal mild macrovacuolar degeneration of hepatocytes, and mild multifocal periportal lymphoplasmacytic inflammatory infiltrate. Small intestine with multifocal mild lymphoplasmacytic enteritis. Lung with multifocal mild presence of basophilic granular foreign body containing numerous diatoms of variable forms in air spaces of parabronchi, diffuse moderate passive congestion, and moderate granulocytic leukocytosis. Kidney with tubular calcification, moderate congestion. Trachea with osseous metaplasia of cartilaginous rings. No alterations were found in heart, gonads, muscle, skin, and thyroid.

118067	Manx Shearwater (*P. puffinus*)	F	J	cachectic	Cardiac atrophy. Congested ventricular mucosa and presence of parasites. Pectoral muscle with atrophy. Millimetric abrasive lesions on the proximal phalanx of the second digit of the left hind limb. Pulmonary edema. Presence of multifocal and moderate firm blackened or whitish parasite nodules in renal parenchyma.	Proventriculus with presence of nematode parasite near cut section. Liver with mild hepatocyte atrophy. No alterations were found in brain, heart, stomach, gonads, intestines, muscle, skin, and lung.
